# Acute Ischemic Stroke Hospital Admissions, Treatment, and Outcomes in Poland in 2009–2013

**DOI:** 10.3389/fneur.2018.00134

**Published:** 2018-03-13

**Authors:** Kamil Chwojnicki, Danuta Ryglewicz, Bogdan Wojtyniak, Paweł Zagożdżon, Anna Członkowska, Tadeusz Jędrzejczyk, Bartosz Karaszewski, Grzegorz Kozera, Marek Gierlotka, Majid Ezzati, Tomasz Zdrojewski

**Affiliations:** ^1^Department of Neurology, Medical University of Gdańsk, Gdańsk, Poland; ^2^Department of Neurology, Polish Institute of Psychiatry and Neurology, Warsaw, Poland; ^3^Polish Institute of Public Health, Warsaw, Poland; ^4^Department of Hygiene and Epidemiology, Medical University of Gdańsk, Gdańsk, Poland; ^5^Department of Public Health, Medical University of Gdańsk, Gdańsk, Poland; ^6^Department of Neurology, Ludwik Rydygier Collegium Medicum of Nicolaus Copernicus University, Bydgoszcz, Poland; ^7^Department of Cardiology, Department of Cardiovascular Diseases, Medical University of Silesia, Silesian Centre for Heart Diseases, Zabrze, Poland; ^8^Faculty of Medicine, School of Public Health, Imperial College of London, London, United Kingdom; ^9^Department of Arterial Hypertension and Diabetology, Medical University of Gdańsk, Gdańsk, Poland

**Keywords:** ischemic stroke, hospitalization, mortality, thrombolysis, outcome

## Abstract

**Introduction:**

Ischemic stroke (IS) still constitutes a serious problem for public health worldwide. The data on its burden in Poland before 2009 is limited and came only from a few metropolitan areas.

**The aims of the study were:**

To assess temporal trends in the hospital admissions, treatment, and outcomes of IS in Poland in 2009–2013, to identify risk factors for IS mortality and to compare the results with other countries.

**Methods:**

The data from the Polish Stroke Registry were analyzed. The data concerned all subjects hospitalized due to IS (classified according to the ICD10 classification as I63.0-I63.9) as primary diagnosis in Poland in 2009–2013. Temporal trends in treatment and outcome were analyzed. Hospital admissions rates as well as case fatality and 12-month mortality rates were calculated.

**Results:**

Altogether, 360,556 patients (47.5% of males) were hospitalized due to IS in Poland in 2009–2013. The median of age was 75 years, IQR 18 (Women 78, IQR 14 vs. Men 70, IQR 17; *p* < 0.001). The hospital admissions age-standardized annual rate for IS in Poland in 2013 was 8% lower than in 2009 (169 vs. 157/100,000; *p* for trend < 0.001). In-hospital case fatality has slightly decreased (from 13.6% in 2009 to 12.9% in 2013; *p* for trend < 0.001). One-year posthospital mortality rate has not changed (19.3% in 2009 and 2013). The percentage of IS subjects treated with intravenous thrombolysis was low but increased from 1.7% in 2009 to 6.3% in 2013 (*p* for trend <0.001).

**Conclusion:**

Since 2009, Poland has had national epidemiological data on the hospital admissions, treatment, and outcomes in IS. The data indicate a slow improvement of in-hospital survival and suggest the need for better stroke prevention and further dissemination of reperfusion therapy.

## Introduction

Ischemic stroke (IS) is one of the major clinical and social problems in the world. It is the second cause of death after coronary heart disease and the main cause of disability among adults (1–3). According to the Global Burden of Disease study, in the years 1990–2010, around 70% of all strokes took place in low- and middle-income countries (LMIC), with Poland included until 2008 (3). Like the other countries of Central and Eastern Europe, Poland belongs to the so-called “high cardiovascular risk group” (4).

Global stroke incidence decreased over the past three decades by 12% in high-income countries, whereas in LMIC, it increased by 12% (3). Until the year 2009, data on IS incidence in Poland were available only for a few metropolitan areas (5–11). Data on IS mortality were more extensive and showed only a small declining trend (12).

The research objectives of this study included the evaluation of hospital admissions, treatment methods, and outcomes related to IS in the years 2009 (the year of transition of Poland from LIMC to high income countries group)—2013. Additional goals were the identification of IS mortality risk factors and comparisons with other countries.

## Materials and Methods

### Source of Data

Data of patients hospitalized due to IS were obtained from the Polish National Stroke Registry (Pol-Stroke Registry). The registry was set up in 2007 in cooperation with the National Health Fund (NHF) under the National Programme for Counteracting Cardiovascular Diseases—Polkard (13). The Pol-Stroke Registry is coordinated by the Polish Institute of Psychiatry and Neurology.

A decision was made to analyze data from 2009 onward because in Poland, a network of stroke units (SU) reporting to the Pol-Stroke Registry had already functioned and an integrated IT medical data collection system was introduced in the NHF in 2009. Otherwise in 2009, the NHF started to refund intravenous recombinant tissue plasminogen activator (rt-Pa) treatment of IS on a national level (before 2009 rt-Pa in IS had only been implemented by individual stroke centers and financed by the Polkard program) (12).

The Pol-Stroke Registry includes all Polish public hospitals, irrespective of their level (1,019 hospitals with 170 SU in 2013), because NHF is the only health insurer in Poland for in-hospital procedures.

The Pol-Stroke Registry database contains demographic data, as well as information on the time and place of hospitalization, etiology of stroke, recurrence of stroke, performed neuroimaging, applying of rt-Pa treatment and mortality. These data are transferred to the NHF by hospital departments, and the NHF refunds the costs of hospitalization on this basis.

In the Pol-Stroke Registry, the WHO stroke definition is used (13); additionally, a diagnosis of IS has to be confirmed by the exclusion of intracranial hemorrhage in neuroimaging (brain CT or MRI). The classification of stroke in the registry is based on the International Statistical Classification of Diseases and Related Health Problems 10th Revision (ICD10). Etiology of stroke is defined on the basis of the TOAST classification (14).

In this article, the analysis included patients aged 18 or older, hospitalized in the years 2009–2013, classified according to the ICD10 as I63.0–I63.9. In case of two or more hospitalizations in the study period, only the first one was taken into account. The number of patients diagnosed with I64 was also specified, but they were excluded from further analyses due to classification difficulties (a lack of brain imaging examination in this group). Patients with no data on follow-up (0.5%) were also excluded from the study.

On the basis of data obtained from the Polish Stroke Registry, the annual hospital admissions rates as well as in-hospital case fatality and 12-month mortality rates were calculated, both crude and standardized by age. A direct method was used to standardize the rates by age. A standard age structure was adopted, the so-called “WHO Standard Population,” identical for men and women, applied by the World Health Organization (15).

Corresponding standardized annual IS mortality rates for selected Eastern-Central Europe countries were obtained from European Health for All Database (HFA-DB) (16).

Stroke death predictors were evaluated for the hospitalization period and in the long-term observation (12 months). Precise data on the dates of deaths were obtained from the NHF (in-hospital deaths) and Central Statistical Office (posthospital deaths). Deaths in Poland are recorded in these institutions on the basis of a death certificate issued by hospital, general practitioner, or doctor from emergency service.

The access to the Polish Stroke Registry database is possible on demand (by contact with corresponding author).

### Statistical Analysis

Simple associations of continuous variables were performed using the *t*-test (normality of data was tested with the Shapiro–Wilk test). Chi-square test was applied in the case of analyses of categorical variables. The multivariate Cox regression models were used to identify the variables predicting death in the acute phase of IS (hospitalization period) as well as in the long-term follow-up (12 months). In this case, hazard ratios, and 95% confidence intervals (CIs) were generated. *p-*Values were two-tailed, and a value of <0.05 was considered to be statistically significant. The variable entry criterion to the Cox model was set to the significance level of *p* ≤ 0.1, and the variable retention criterion to *p* < 0.05. Due to the lack of information on the incidence of previous IS in 10% of the subjects, the multiple imputation method for arbitrary pattern of missingness was used to replace the missing values (fully conditional specification discriminant model). The significance of the time trends in the studied years was evaluated using the Cochran–Armitage test (categorical variables) or the Jonckheere–Terpstra test (continuous variables). All statistical analyses were performed using SAS^®^ 9.4 Foundation software (SAS Institute, Cary, NC, USA).

## Results

### Hospitalizations and Diagnoses

Altogether, 360,556 patients (47.5% of males), coded with I63.0–I63.9 according to the ICD10 classification, were hospitalized in Poland in the years 2009–2013. The number of hospitalized patients was slightly different in subsequent years. The lowest number of hospitalizations was recorded in 2010, the highest in 2012 (Table 1).

**Table 1:** Diagnoses according to the ICD10 classification made during hospitalization.

Year	ICD10	I63.0*	I63.1	I63.2	I63.3*	I63.4*	I63.5*	I63.6	I63.8	I63.9	I64*	All
2009	*n*	1,985	573	2,345	13,258	11,464	15,387	107	9,724	16,797	4,501	76,141
	%	2.61	0.75	3.08	17.41	15.06	20.21	0.14	12.77	22.06	5.91	100.00

2010	*n*	1,797	692	2,762	13,485	12,314	15,271	90	9,099	14,990	2,645	73,145
	%	2.46	0.95	3.78	18.44	16.84	20.88	0.12	12.44	20.49	3.62	100.00

2011	*n*	1,578	866	3,151	14,344	13,658	15,389	96	8,930	14,476	2,636	75,124
	%	2.10	1.15	4.19	19.09	18.18	20.48	0.13	11.89	19.27	3.51	100.00

2012	*n*	1,539	746	2,813	15,471	14,922	14,963	110	9,377	13,201	2,531	75,673
	%	2.03	0.99	3.72	20.44	19.72	19.77	0.15	12.39	17.44	3.34	100.00

2013	*n*	1,385	743	2,355	16,455	15,438	13,356	150	9,159	13,747	2,320	75,106
	%	1.84	0.99	3.14	21.91	20.55	17.78	0.20	12.19	18.30	3.09	100.00

*p for trend 2009–2013 < 0.001.

**Figure 1:**
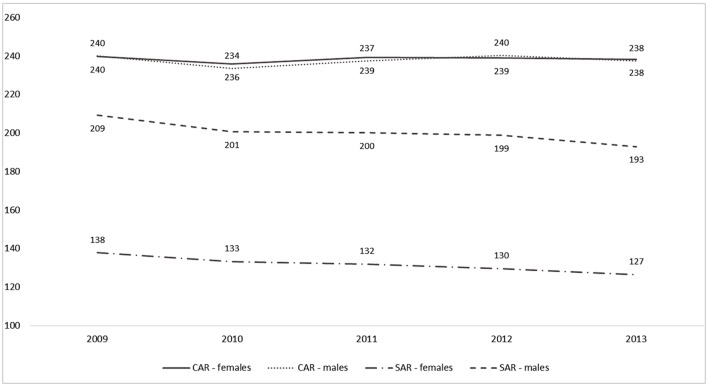
Crude (CAR) and age-standardized (SAR) annual hospital admissions rates (per 100,000) for ischemic stroke in Poland in 2009–2013.

Annual crude (CAR) and standardized by age (SAR) hospital admissions rates of IS patients in Poland, calculated on the basis of the data obtained from Pol-Stroke Registry, are presented in Figure 1 and Table S1 in Supplementary Material. While CAR for both sexes was very similar and did not change in the examined period, SAR significantly decreased (in 2013, SAR was 8% lower compared to 2009—*p* for trend < 0.001).

The highest percentage (54%) of diagnoses according to ICD10 classification was constituted by the least precise diagnoses: I63.5—cerebral infarction due to unspecified occlusion or stenosis of unspecified cerebral artery, I63.8—other cerebral infarction, I63.9—cerebral infarction, unspecified. In the analyzed period, a significant increase was recorded in the diagnoses of embolic strokes (I63.1 + I63.4). Only occasionally, a stroke caused by venous thrombosis was diagnosed (I63.6—0.15%). There was a twofold decrease (from 5.9 to 3.1%) in the number of I64 diagnoses (stroke, not specified as hemorrhage or infarction) in the years 2009–2013.

Among the subjects diagnosed with I63.0–I63.9, the median age was 75 years, IQR 57–93 (women 78, 64–92 vs. men 70, 53–97; *p* < 0.001). The median age of hospitalized patients was not changing in the analyzed period.

In three quarters of cases, the patients were hospitalized due to their first stroke (Table 2). The 1-year IS recurrence was reduced from 8.6% in 2009 to 7.7% in 2013 (*p* for trend < 0.001).

**Table 2:** Baseline characteristics of hospitalized subjects coded with I63.0–I63.9.

Demographics	2009	2010	2011	2012	2013
Hospitalizations due to IS (*N*)	71,640	70,500	72,488	73,142	72,786
Males (*N*; %)	34,001; 47.46	33,283; 47.21	34,392; 47.45	34,940; 47.77	34,631; 47.58
Age (Mean ± SD)*	72.42 ± 11.84	72.46 ± 12.00	72.73 ± 12.03	72.80 ± 12.07	73.00 ± 12.11
First IS (*N*; %)*	45,891; 76.70	48,873; 77.24	50,411; 77.25	51,232; 77.64	51,301; 78.08
Cardiac IS (*N*; %)*	12,037; 16.80	13,006; 18.45	14,524; 20.04	15,668; 21.42	16,181; 22.23
Treatment in SU (*N*; %)*	60,189; 84.02	60,939; 86.44	62,810; 86.65	64,809; 88.61	65,177; 89.55
IVT treatment (*N*; %)*	1,229; 1.72	1,830; 2.60	2,975; 4.10	4,023; 5.50	4,608; 6.33
10 days case fatality (%)*	10.45	10.36	10.47	10.17	9.88
In-hospital case fatality (%)*	13.56	13.56	13.50	13.08	12.86
1-month mortality (%)*	17.09	16.64	16.63	16.26	16.36
3-month mortality (%)*	23.97	23.52	23.46	23.24	23.35
12-month mortality (%)*	32.82	33.03	32.52	32.31	32.15
12-month posthospital mortality (%)	19.26	19.47	19.02	19.23	19.29

*p for trend 2009–2013 < 0.01.

*SU, stroke unit; IS, ischemic stroke; IVT, intravenous thrombolysis*.

### Treatment

The percentage of patients hospitalized in SU during the analyzed period was relatively high (more than 80%), and it increased every year (in total by 5.5% between the years 2009 and 2013).

The intravenous thrombolysis was rarely used, and yet, a percentage of patients treated with rt-Pa increased with time (from 1.7% in 2009 to 6.3% in 2013; *p* for trend < 0.01). The thrombolyzed patients were younger. They were also more often hospitalized in SU. The percentage of patients with first stroke and cardiogenic stroke was significantly higher in the thrombolyzed group than in the non-thrombolyzed group (Table 3).

**Table 3:** Baseline characteristics of IVT and no-IVT treated stroke subjects.

			Age	Age >80		Males		First IS		Cardiac IS		SU	

Year		Total	Mean ± SD	*N*	%	*N*	%	*N*	%	*N*	%	*N*	%
2009	All	71,640	72.4 ± 11.8	19,919	27.8	34,001	47.5	45,891	76.7	12,037	16.8	60,189	84.0
	IVT	1,229 (1.7%)	68.4 ± 12.2	178	14.5	643	52.3	852	84.0	425	34.6	1,145	93.2
	No IVT	70,411 (98.3%)	72.5 ± 11.8	19,741	28.0	33,358	47.4	45,039	76.6	11,612	16.5	59,044	83.9
	*p*	<0.01	<0.01	<0.01		<0.01		<0.01		<0.01		<0.01	

2010	All	70,500	72.5 ± 12.0	20,251	28.7	33,283	47.2	48,873	77.2	13,006	18.4	60,939	86.4
	IVT	1,830 (2.6%)	68.7 ± 12.2	275	15.0	963	52.6	1,472	86.3	716	39.1	1,752	95.7
	No IVT	68,670 (97.4%)	72.6 ± 12.0	19,976	29.1	32,320	47.1	47,401	77.0	12,290	17.9	59,187	86.2
	*p*	<0.01	<0.01	<0.01		<0.01		<0.01		<0.01		<0.01	

2011	All	72,488	72.7 ± 12.0	22,006	30.4	34,392	47.4	50,411	77.2	14,524	20.0	62,810	86.6
	IVT	2,975 (4.1%)	69.5 ± 12.4	577	19.4	1,528	51.4	2,385	86.9	1,307	43.9	2,911	97.8
	No IVT	69,513 (95.9%)	72.9 ± 12.0	21,429	30.8	32,864	47.3	48,026	76.8	13,217	19.0	59,899	86.2
	*p*	<0.01	<0.01	<0.01		<0.01		<0.01		<0.01		<0.01	

2012	All	73,142	72.8 ± 12.1	22,555	30.8	34,940	47.8	51,232	77.6	15,668	21.4	64,809	88.6
	IVT	4,023 (5.5%)	70.2 ± 12.3	882	21.9	2,067	51.4	3,160	85.8	1,837	45.7	3,968	98.6
	No IVT	69,119 (94.5%)	73.0 ± 12.0	21,673	31.4	32,873	47.6	48,072	77.2	13,831	20.0	60,841	88.0
	*p*	<0.01	<0.01	<0.01		<0.01		<0.01		<0.01		<0.01	

2013	All	72,786	73.0 ± 12.1	23,103	31.7	34,631	47.6	51,301	78.1	16,181	22.2	65,177	89.6
	IVT	4,608 (6.3%)	69.8 ± 12.6	1,036	22.5	2,394	51.9	3,724	87.1	2,328	50.5	4,608	100.0
	No IVT	68,178 (93.7%)	73.2 ± 12.0	22,067	32.4	32,237	47.3	47,577	77.4	13,853	20.3	60,569	88.8
	*p*	<0.01	<0.01	<0.01		<0.01		<0.01		<0.01		<0.01	

*SU, stroke unit; IS, ischemic stroke; IVT, intravenous thrombolysis*.

### Mortality

On average, 1 in 10 hospitalized persons died within the first 10 days after admission. In-hospital case fatality was 13.6%. For 1, 3-, and 12-month follow-up mortality was 17, 23, and 32%, respectively. In subsequent years, a minimal decrease of in-hospital case fatality was observed; however, posthospital mortality has not changed (Table 2).

**Figure 2:**
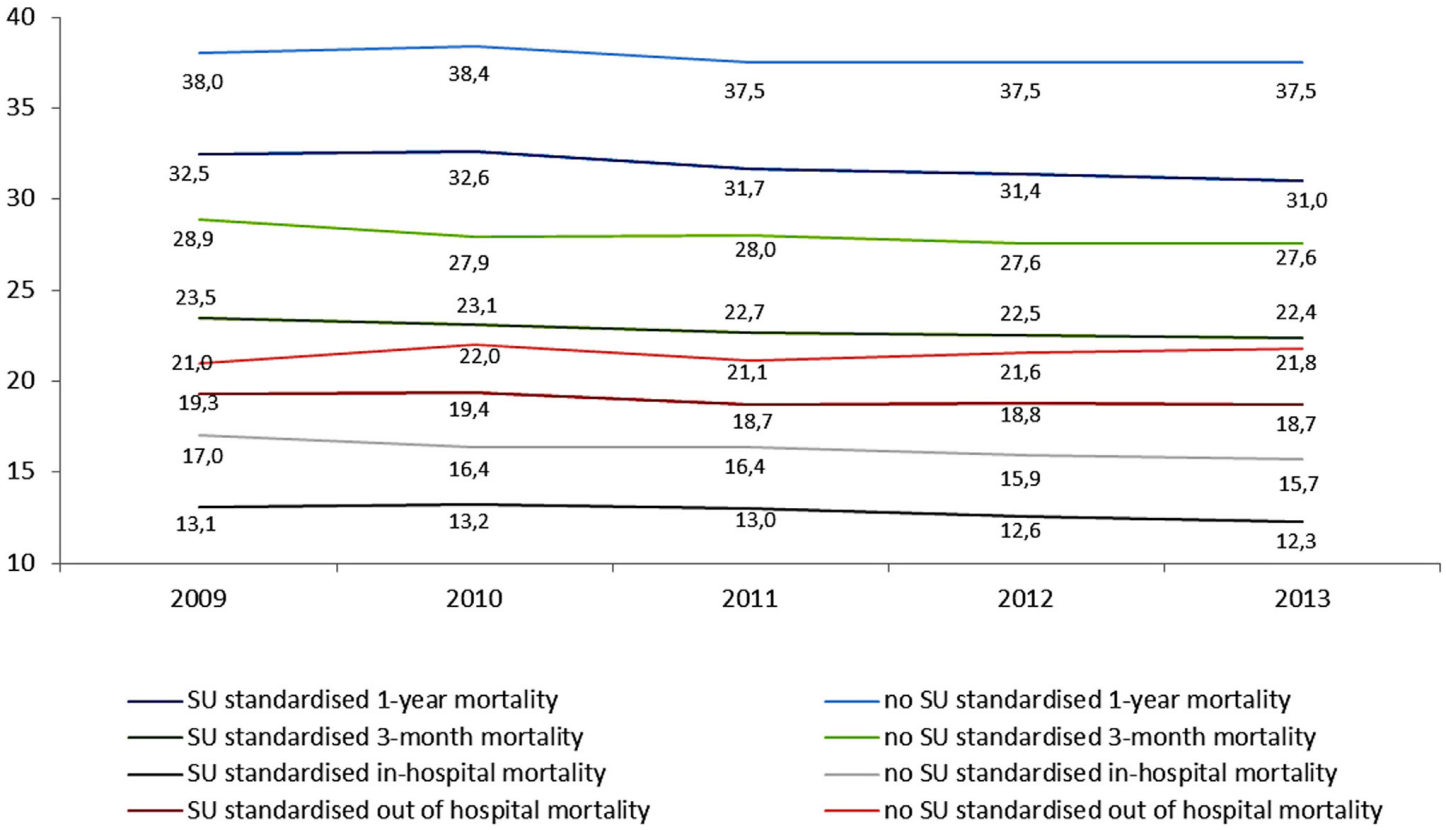
Standardized death rates for subjects hospitalized due to ischemic stroke in 2009–2013 with regard to time from stroke and place of treatment.

Annual standardized death rates (SDR) for patients hospitalized due to IS are presented in Figure 2 and Table S1 in Supplementary Material. Only a small declining trend for SDR was observed for hospitalization periods and SU. Overall, SDR has not changed from 2009 to 2013.

In the multivariate analysis (Cox proportional hazards model built from chosen variables presented in Table 4), a small yet systematic positive effect for survival probability was noted, especially for first stroke patients and those hospitalized in SU Female sex, receiving thrombolytic treatment and developing stroke in the last years rather than previous ones, also constituted positive prognostic factors. Older age and cardiogenic stroke were negative prognostic factors. These associations were more clear for in-hospital than for posthospital period (Table 5).

**Table 4:** Characteristics of hospitalized IS population by the time of death.

Year	Death period	Total	Age	Age >80		Males		First IS		Cardiac IS		SU		IVT	
			Mean ± SD	*N*	%	*N*	%	*N*	%	*N*	%	*N*	%	*N*	%
2009	All	71,640	72.4 ± 11.8	19,919	27.8	34,001	47.5	45,891	76.7	12,037	16.8	60,189	84.0	1,229	1.7
	
	Death up to 10 days	Yes	77.7 ± 10.3	3,379	45.1	3,180	42.5	4,521	75.5	1,516	20.2	5,850	78.1	146	2.0
		No	71.8 ± 11.9	16,540	25.8	30,821	48.0	41,370	76.8	10,521	16.4	54,339	84.7	1,083	1.7
		*p*	<0.01	<0.01		<0.01		<0.01		<0.01		<0.01		0.01	
	
	Death up to 3 months	Yes	78.3 ± 9.9	8,187	47.7	7,076	41.2	10,202	73.8	3,619	21.1	13,676	79.6	285	1.7
		No	70.6 ± 11.8	11,732	21.5	26,925	49.4	35,689	77.6	8,418	15.5	46,513	85.4	944	1.77
		*p*	<0.01	<0.01		<0.01		<0.01		<0.01		<0.01		0.5	
	
	Death up to 1 year	Yes	78.0 ± 9.9	10,867	46.2	9,775	41.6	13,948	72.8	4,837	20.6	18,924	80.5	375	1.6
		No	69.7 ± 11.8	9,052	18.8	24,226	50.3	31,943	78.6	7,200	15.0	41,265	85.8	854	1.8
		*p*	<0.01	<0.01		<0.01		<0.01		<0.01		<0.01		0.08	
	
2010	All	70,500	72.5 ± 12.0	20,251	28.7	33,283	47.2	48,873	77.2	13,006	18.4	60,939	86.4	1,830	2.6
	
	Death up to 10 days	Yes	78.0 ± 10.3	3,423	46.9	3,019	41.4	4,824	75.2	1,671	22.9	5,969	81.8	252	3.4
		No	71.8 ± 12.0	16,828	26.6	30,264	47.9	44,049	77.5	11,335	17.9	54,970	87.0	1,578	2.5
		*p*	<0.01	<0.01		<0.01		<0.01		<0.01		<0.01		<0.01	
	
	Death up to 3 months	Yes	78.6 ± 10.0	8,169	49.3	6,732	40.6	10,616	73.4	3,736	22.5	13,742	82.9	430	2.6
		No	70.6 ± 11.9	12,082	22.4	26,551	49.2	38,257	78.4	9,270	17.2	47,197	87.5	1,400	2.6
		*p*	<0.01	<0.01		<0.01		<0.01		<0.01		<0.01		1.0	
	
	Death up to 1 year	Yes	78.1 ± 10.0	11,023	47.3	9,576	41.1	14,830	72.4	5,171	22.2	19,420	83.4	558	2.4
		No	69.7 ± 11.9	9,228	19.6	23,707	50.2	34,043	79.6	7,835	16.6	41,519	87.9	1,272	2.7
		*p*	<0.01	<0.01		<0.01		<0.01		<0.01		<0.01		0.02	
	
2011	All	72,488	72.7 ± 12.0	22,006	30.4	34,392	47.4	50,411	77.2	14,524	20.0	62,810	86.6	2,975	4.1
	
	Death up to 10 days	Yes	78.4 ± 10.5	3,750	49.4	3,061	40.3	5,149	76.9	1,962	25.8	6,217	81.9	349	4.6
		No	72.1 ± 12.0	18,256	28.1	31,331	48.3	45,242	77.3	12,562	19.4	56,593	87.2	2,626	4.0
		*p*	<0.01	<0.01		<0.01		0.52		<0.01		<0.01		0.02	
	
	Death up to 3 months	Yes	78.9 ± 10.0	8,766	51.6	6,778	39.9	11,051	74.1	4,365	25.7	14,091	82.9	665	3.9
		No	70.8 ± 11.9	13,240	23.9	27,614	49.8	39,360	78.2	10,159	18.3	48,719	87.8	2,310	4.2
		*p*	<0.01	<0.01		<0.01		<0.01		<0.01		<0.01		0.15	
	
	Death up to 1 year	Yes	78.5 ± 10.1	11,780	50.0	9,530	40.4	15,202	73.1	5,894	25.0	19,688	83.5	871	3.7
		No	70.0 ± 11.9	10,226	20.9	24,862	50.8	35,209	79.2	8,630	17.6	43,122	88.2	2,104	4.3
		*p*	<0.01	<0.01		<0.01		<0.01		<0.01		<0.01		<0.01	
	
2012	All	73,142	72.8 ± 12.1	22,555	30.8	34,940	47.8	51,232	77.6	15,668	21.4	64,809	88.6	4,023	5.5
	
	Death up to 10 days	Yes	78.4 ± 10.4	3,754	50.5	3,105	41.8	5,051	77.0	2,041	27.4	6,218	83.6	483	6.5
		No	72.1 ± 12.0	18,801	28.6	31,835	48.4	46,181	77.7	13,627	20.7	58,591	89.2	3,540	5.4
		*p*	<0.01	<0.01		<0.01		0.22		<0.01		<0.01		<0.01	
	
	Death up to 3 months	Yes	79.1 ± 10.0	8,968	52.8	6,856	40.3	11,046	74.4	4,599	27.0	14,467	85.1	955	5.6
		No	70.9 ± 12.0	13,587	24.2	28,084	50.0	40,186	78.6	11,069	19.7	50,342	89.7	3,068	5.5
		*p*	<0.01	<0.01		<0.01		<0.01		<0.01		<0.01		0.44	
	
	Death up to 1 year	Yes	78.7 ± 10.1	12,003	50.8	9,679	41.0	15,343	73.8	6,202	26.2	20,219	85.6	1,230	5.2
		No	70.0 ± 11.9	10,552	21.3	25,261	51.0	35,889	79.4	9,466	19.1	44,590	90.1	2,793	5.6
		*p*	<0.01	<0.01		<0.01		<0.01		<0.01		<0.01		0.02	
	
2013	All	72,786	73.0 ± 12.1	23,103	31.7	34,631	47.6	51,301	78.1	16,181	22.2	65,177	89.6	4,608	6.3
	
	Death up to 10 days	Yes	78.6 ± 10.4	3,731	51.9	2,812	39.1	4,910	77.2	1,984	27.6	6,125	85.1	427	5.9
		No	72.3 ± 12.1	19,372	29.5	31,819	48.5	46,391	78.2	14,197	21.6	59,052	90.0	4,181	6.4
		*p*	<0.01	<0.01		<0.01		0.08		<0.01		<0.01		0.15	
	
	Death up to 3 months	Yes	79.5 ± 12.0	9,229	54.3	6,695	39.4	11,046	74.4	4,692	27.6	14,681	86.4	916	5.4
		No	71.0 ± 12.0	13,874	24.9	27,936	50.1	40,186	78.6	11,489	20.6	50,496	90.5	3,692	6.6
		*p*	<0.01	<0.01		<0.01		<0.01		<0.01		<0.01		<0.01	
	
	Death up to 1 year	Yes	79.1 ± 10.1	12,281	52.5	9,398	40.2	15,283	74.2	6,344	27.1	20,276	86.6	1,176	5.0
		No	70.1 ± 11.9	10,822	21.9	25,233	51.1	36,018	79.9	9,837	19.9	44,901	90.9	3,432	7.0
		*p*	<0.01	<0.01		<0.01		<0.01		<0.01		<0.01		<0.01	

*SU, stroke unit; IS, ischemic stroke; IVT, intravenous thrombolysis*.

**Table 5:** Results of Cox proportional hazards model for death in consecutive time intervals.

	In-hospital mortality			Posthospital mortality (up to 1 year)			Death up to 1 year (overall)		
			
Parameter	HR	95% CI	*p*	HR	95% CI	*p*	HR	95% CI	*p*
Sex (males vs. females)	0.978	0.975–0.981	<0.001	0.970	0.966–0.973	<0.001	0.963	0.960–0.967	<0.001
Age (per 10 years more)	1.128	1.126–1.131	<0.001	1.109	1.107–1.111	<0.001	1.077	1.076–1.078	<0.001
IVT (yes/no)	0.927	0.911–0.944	<0.001	0.976	0.958–0.994	0.009	0.957	0.948–0.965	<0.001
First IS (yes/no)	0.880	0.877–0.884	<0.001	0.890	0.887–0.894	<0.001	0.918	0.914–0.922	<0.001
Cardiac IS (yes/no)	1.083	1.073–1.0.93	<0.001	1.060	1.055–1.065	<0.001	1.038	1.033–1.043	<0.001
Stroke unit (yes/no)	0.898	0.894–0.902	<0.001	0.917	0.912–0.922	<0.001	0.928	0.923–0.934	<0.001
Year of IS (per 1 year later)	0.931	0.930–0.932	<0.001	0.923	0.922–0.924	<0.001	0.917	0.915–0.918	<0.001

*HR, hazards ratio; CI, confidence interval; IS, ischemic stroke; IVT, intravenous thrombolysis*.

*Variables for the multivariate analysis were selected on the basis of **Table 4***.

## Discussion

This article shows comprehensive national data on hospitalizations and outcome of IS in Poland in the years 2009–2013. The epidemiologic data presented are more complex than before 2009, since they were collected in an integrated manner and according to the established criteria (apart from the IS diagnosis criterion according to WHO, reporting required also: performance of brain imaging—CT or MRI and identification of etiology).

Data presented in the article indicate that IS is still a very significant problem for the Polish medical care and health policy.

In our analysis, we assessed “hospital admissions” rates and not “incidence” rates (there are no Polish data on non-hospitalized strokes). It seems, however, that in Poland, the actual difference between these rates may not be large. In the case of IS, there is very little sudden death in the pre-hospital period (unlike coronary heart disease and hemmorhagic or subarachnoid hemorrhage). In Poland, there is a rule that every patient with stroke requires hospital treatment. Thorough hospital diagnostics of stroke is additionally financed, hence a small number of false-negative cases. From the other site, the number of non-hospitalized strokes can be probably larger in oldest ages but we have no Polish data on that.

Standardized hospital admissions rates for IS in Poland in the period 2009–2013 are still high despite an obvious downward trend. They significantly exceeds standardized incidence rate for LMIC for the years 2000–2008 and EU Member States from before 2004 (EU-15) (3).

**Figure 3:**
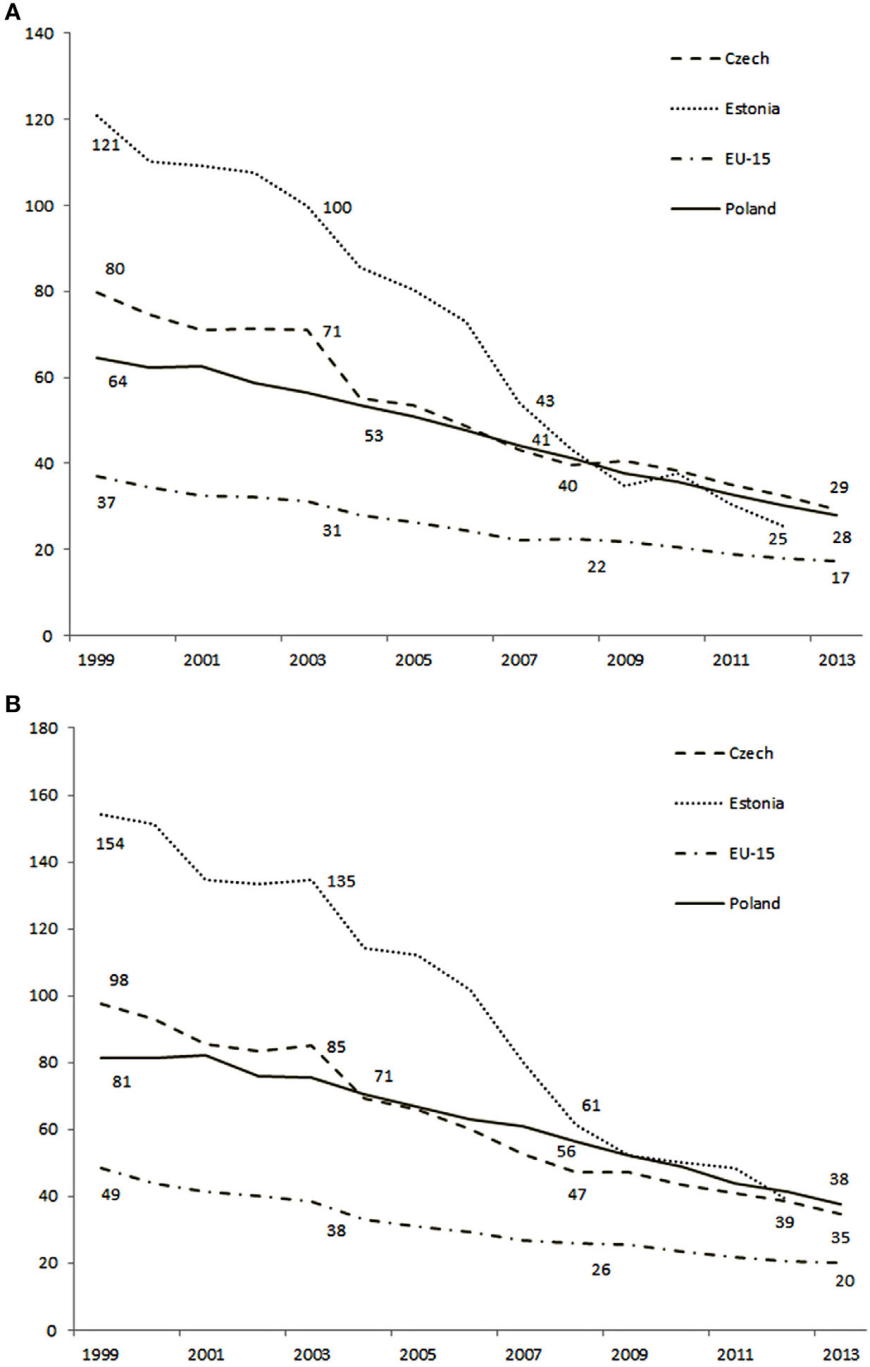
Standardized annual mortality rates (per 100,000) in selected Eastern-Central Europe countries: females **(A)** and males **(B)**, respectively.

Ischemic stroke death rates are still high compared with the EU-15. As in the case of SAR, SDR in Poland also exceeds the average value for the EU-15 by more than twice for men and 1.5 times for women. Compared to the EU-28 average, SDR in Poland was about 22% higher (HFA-DB 2014). The number of healthy life years lost in Poland is at least 2.7 times higher than the EU-15 average (2012). A decrease in SDR vs. time is visible, yet very slow. With regard to SDR, Poland is similar to other countries of the region, such as, for example, the Czech Republic or Estonia; however, it can be seen that the fastest rate of decrease in the SDR since 2000 was recorded in Estonia (Figure 3) (16). IS death rates in Russia and Ukraine are ca. three times higher than in Poland but the analysis of trends is hindered due to incomplete data (16).

What is worth noting is the fact that the 10-days’ case fatality essentially does not differ from the average for Western European countries, which suggests a good quality of hospital care and this may be due to the development of SU (1). In the 5-year analyzed period, the number of SU increased by more than 50%. In 2013, their number was 170 (1 SU/220,000 people), in 2007, it was only 111 (1 SU/340,000). In addition, in 2013, more than 90% of SU declared the fulfillment of criteria of type A units (eligible for implementing systemic thrombolysis according to the criteria of the National Stroke Prevention and Treatment Program and EUSI guidelines), whereas in 2007, they constituted only 55% (17). In 2013, 86% of stroke hospitalizations took place in the SU, which should be considered as a good implementation of the European recommendations for stroke treatment. These organizational changes and gradual improvement in treatment are most likely responsible for decreased trends in total early case-fatality in IS.

Interestingly, over time, the impact of the development of new SU on the mortality from IS seems to be getting smaller. There was a greater decrease of the relevant indicators in 1995–2006 than after 2006 (18).

The 12-month death rate of patients hospitalized in Poland in 2009–2013, at a level of 32%, should be considered as very high compared to the USA or Western European countries. In our study, it is comparable to Hungary (31%) and Scotland (28%), but significantly higher than in the Netherlands (23%), Finland (21%), Sweden (20%), and Italy (16%) (19). Posthospital mortality in IS had changed minimally in Poland over the decade. This leads to conclusion, which progress applies mainly to the acute phase of IS. Thus, further actions in poststroke prevention should be addressed in near future.

An important factor is still the insufficient number of conducted diagnostics concerning causes of IS. A number of recognizable cerebral venous thromboses at the maximum level of 0.2% is at least 2.5 times lower than assumptions of, for example, clinical research data (20). One should, however, note that, as a matter of principle, data from clinical tests are of a better quality than registration data. This is due to the fact that clinical tests are conducted in experienced clinical centers.

In Poland, so-called strokes due to “other” or “unspecified” causes (I63.5,8,9—55% in 2009 and 48% in 2013) are dominant diagnoses in SU. These diagnoses are more common in Poland than, for example, in Germany (54 vs. 44%) (21). On the other hand, the percentage of cardiogenic strokes, first of all, increases and, second, is at a similar level to other registries and epidemiological studies (22).

Frequent low-precision diagnoses, such as I63.5,8,9, I64 are a problem that undermines the fulfillment of type A criteria by SU in Poland. It may result from the method of financing hospitalization due to cerebral stroke in Poland—it is the same in the case of codes ranging from I60.0 to I64: a precision is irrelevant here, a consequence is most surely the lack of due diagnostic diligence.

Another significant problem relates to a specific therapy—despite a permanent growth in the number of thrombolyzed persons, its percentage is still insufficient (only ca. 6.3% in 2013), whereas it is one of the most important quality indicators of IS treatment. For comparison, in Germany, the percentage of thrombolyzed subjects with IS was 9% in 2009 (23) and 15% in 2014 (data from 12th International Symposium on Thrombolysis Thrombectomy and Acute Stroke Therapy, Heidelberg 2014). In the Netherlands, the percentage of persons treated with rt-Pa increased from 6.4% in 2005 to 14.6% in 2012 (24). Poland is ranked slightly below other countries of the region—for instance, in the Czech Republic, it reached 7% already in 2011 (25). However, in Poland, the annual growth in the number of thrombolyzed persons is observed. A further growth in the percentage of patients undergoing rt-Pa therapy is anticipated in the future. According to unpublished NHF data, the percentage of thrombolyzed persons in Poland amounted to 8% in 2014 and in 2015 it was already 10%. This means a six times growth as compared with 2009. For the sake of comparison, in the USA, there was only a twofold increase in the percentage of thrombolyzed persons in the period 2005–2011 (26).

### Limitations

The analysis of national data is based on the group of hospitalized patients, and it is known that ca. 11% of patients with stroke do not reach hospitals (27). This is, however, not only a Polish limitation. In the Pol-Stroke registry database, there are no strokes that are hospitalized in private hospitals. Such cases are very rare in Poland, since private hospitals do not provide the treatment of stroke. Even in the case of an iatrogenic stroke, the patient is usually transferred to a public unit. The Pol-Stroke Registry does not contain much important information, such as functional status before and after stroke and onset-to-door as well as door-to-needle time. On the basis of data from the registry, it was impossible to determine how the increase in the percentage of persons undergoing thrombolytic treatment was affected by the change in recommendations concerning extending the time window to 4.5 h (28). From the above reasons, the modification of data collected under the registry would be very desirable. One should take account of limitations resulting from using ICD10 and TOAST classifications in the register in the context of diagnosing rare causes of IS. The quality of coding varies from country to country, potentially affecting the value of case ascertainment. The error range related to the encoding of stroke type in the Pol-Stroke registry is difficult to estimate. The NHF controls annually about 1% of public hospitals (including 1–2 SU). In some situations, however, the institution cannot verify the correctness of the diagnosis using its control tools (for example, it can easily distinguish between IS and haemorrhagic stroke, but not stroke mimics). It is difficult to compare case-fatality rates between countries since there is no consideration of the demographic structure of the population. Only SDR are relevant in that purpose.

To sum up, Poland has reliable epidemiological data on hospitalizations, treatment, and outcomes of IS from 2009. After transition to EU (2004) and to high income country group (2009), the improvement of the epidemiological situation in Poland is still too slow despite intensive development of the SU network (except in-hospital IS mortality, which is similar to “old EU countries”). The data also emphasize the need for further dissemination of thrombolytic therapy. With regular monitoring it will be possible to assess the dynamics of changes that will occur.

## Author Contributions

KC: study design, data interpretation, statistical analysis, and manuscript draft. DR, BW, AC, BK, GK, TJ, MG, and ME: data interpretation, manuscript draft. PZ: statistical analysis; TZ: study design, data interpretation, and manuscript draft.

## Conflict of Interest Statement

The authors declare that the research was conducted in the absence of any commercial or financial relationships that could be construed as a potential conflict of interest.

## References

[1] Feigin VL Lawes CM Bennett DA Barker-Collo SL Parag V. Worldwide stroke incidence and early case fatality reported in 56 population-based studies: a systematic review. Lancet Neurol (2009) 8:355–69. 10.1016/S1474-4422(09)70025-0 19233729

[2] Feigin V Lawes C Bennett D Anderson C. Stroke epidemiology: a review of population-based studies of incidence, prevalence, and case-fatality in the late 20th century. Lancet Neurol (2003) 1:43–53. 10.1016/S1474-4422(03)00266-7 12849300

[3] Feigin VL Forouzanfar MH Krishnamurthi R Mensah GA Connor M Bennett DA Global and regional burden of stroke during 1990–2010: findings from the Global Burden of Disease Study 2010. Lancet (2014) 383:245–54. 10.1016/S0140-6736(13)61953-4 24449944 PMC4181600

[4] Conroy RM Pyorala K Fitzgerald AP Sans S Menotti A De Backer G SCORE project group: estimation of ten-year risk of fatal cardiovascular disease in Europe: the SCORE project. Eur Heart J (2003) 24:987–1003. 10.1016/S0195-668X(03)00114-3 12788299

[5] Czlonkowska A Ryglewicz D Weissbein T Baranska-Gieruszczak M Hier DB A prospective community-based study of stroke in Warsaw, Poland. Stroke (1994) 5:547–51. 10.1161/01.STR.25.3.547 8128505

[6] Ryglewicz D Polakowska M Lechowicz W Broda G Rószkiewicz M Jasinski B Stroke mortality rates in Poland did not decline between 1984 and 1992. Stroke (1997) 28:752–7. 10.1161/01.STR.28.4.752 9099191

[7] Thorvaldsen P Asplund K Kuulasmaa K Rajakangas AM Schroll M Stroke incidence, case fatality, and mortality in the WHO MONICA project. World Health Organization monitoring trends and determinants in cardiovascular disease. Stroke (1995) 26:361–7. 10.1161/01.STR.26.3.361 7886707

[8] The European Registers of Stroke (EROS) Investigators. Incidence of stroke in Europe at the beginning of the 21st century. Stroke (2009) 40:1557–63. 10.1161/STROKEAHA.108.535088 19325154

[9] Heuschmann PU Wiedman S Wellwood I Rudd A Di Carlo A Bejot Y European registers of stroke. Three-month stroke outcome: The European Registers of Stroke (EROS) Investigators. Neurology (2011) 76:159–65. 10.1212/WNL.0b013e318206ca1e 21148118

[10] Słowik A Turaj W Zwolińska G Róg T Dziedzic T Pera J Stroke attack rates and case fatality in the Krakow Stroke Registry. Neurol Neurochir Pol (2007) 41:291–5. 17874336

[11] Sienkiewicz-Jarosz H Głuszkiewicz M Pniewski J Niewada M Członkowska A Wolfe Incidence and case fatality rates of first-ever-stroke – comparison of data from two prospective population-based studies conducted in Warsaw. Neurol Neurochir Pol (2011) 45:207–12. 21866477 10.1016/s0028-3843(14)60073-6

[12] Członkowska A Niewada M Sarzyńska-Dugosz I Kobayashi A Skowrońska M Ten years of stroke programmes in Poland: where did we start? Where did we get to? Int J Stroke (2010) 5:414–6. 10.1111/j.1747-4949.2010.00470.x 20854627

[13] WHO Monica Project Investigators. The World Health Organization MONICA Project (Monitoring trends and determinants in cardiovascular disease). J Clin Epidemiol (1988) 41:105–14. 10.1016/0895-4356(88)90084-4 3335877

[14] Adams HP Bendixen BH Kapelle J Biller J Love BB Gordon DL. Classification of subtype of acute ischemic stroke. Definitions for use in a multicenter clinical trial. TOAST. Trial of Org 10172 in acute stroke treatment. Stroke (1993) 24:35–41. 10.1161/01.STR.24.1.35 7678184

[15] Ahmad OE Boschi-Pinto C Lopez AD Murray CJL Lozano R Inoue M Age Standardization of Rates: A New WHO Standard GPE Discussion Paper Series: No. 31. Geneva: World Health Organization (2000).

[16] World Health Organization Regional Office for Europe. European Health for All Family of Databases. WHO Europe Available from: http://www.euro.who.int/en/data-and-evidence/databases/european-health-for-all-family-of-databases-hfa-db (accessed March 2017).

[17] Sarzyńska-Długosz I Skowrońska M Członkowska A Organization of acute stroke services in Poland – Polish Stroke Unit Network development. Neurol Neurochir Pol (2013) 47:3–7. 10.5114/ninp.2013.32934 23487288

[18] Ryglewicz D Lechowicz W Sienkiewicz-Jarosz H Dynamic changes of stroke care in Poland on the basis of Polish National Stroke Registry (2008–2014). Cerebrovasc Dis (2016) 41:216–7.

[19] Malmivaara A Meretoja A Peltola M Numerato D Heijink R Engelfriet P Comparing ischaemic stroke in six European countries. The EuroHOPE register study. Eur J Neurol (2015) 22:284–91. 10.1111/ene.12560 25196190

[20] Bousser MG Ferro JM. Cerebral venous thrombosis: an update. Lancet Neurol (2007) 6:162–70. 10.1016/S1474-4422(07)70029-7 17239803

[21] Kolominsky-Rabas P Weber M Gefeller O Neundoerfer B Heuschmann PU. Epidemiology of ischemic stroke subtypes according to TOAST criteria. Incidence, recurrence, and long-term survival in ischemic stroke subtypes: a population-based study. Stroke (2001) 32:2735–40. 10.1161/hs1201.100209 11739965

[22] Arboix A Alió J Cardioembolic stroke: clinical features, specific cardiac disorders and prognosis. Curr Cardiol Rev (2010) 6(3):150–61. 10.2174/157340310791658730 21804774 PMC2994107

[23] Scholten N Pfaff H Lehmann HC Fink GR Karbach U. Who does it first? The uptake of medical innovations in the performance of thrombolysis on ischemic stroke patients in Germany: a study based on hospital quality data. Implementation Sci (2015) 10:10. 10.1186/s13012-014-0196-7 25582164 PMC4300164

[24] Scherf S Limburg M Wimmers R Middelkoop I Lingsma H. Increase in national intravenous thrombolysis rates for ischaemic stroke between 2005 and 2012: is bigger better? BMC Neurol (2016) 16:53. 10.1186/s12883-016-0574-7 27103535 PMC4839134

[25] Jackova J Sedova P Brown R Jr Bryndziar T Zvolsky M Bednarik J The high frequency of guideline-approved and guideline-disapproved medication use in stroke and transient ischemic attack. J Stroke Cerebrovasc Dis (2016) 25:2688–93. 10.1016/j.jstrokecerebrovasdis.2016.07.016 27476339

[26] Schwamm LH Ali Syed F Reeves MJ Smith EE Saver JL Messe S Temporal trends in patient characteristics and treatment with intravenous thrombolysis among acute ischemic stroke patients at get with the guidelines–stroke hospitals. Circulation (2013) 6:543–9. 10.1161/CIRCOUTCOMES.111.000095 24046398

[27] National Stroke Foundation. National Stroke Audit – Acute Services Clinical Audit Report 2011. Melbourne: NSF (2011).

[28] The European Stroke Organisation (ESO) Executive Committee. Guidelines for management of ischaemic stroke and transient ischaemic attack 2008. Cerebrovasc Dis (2008) 25:457–507. 10.1159/000131083 18477843

