# Supplementary material for: Acute Ischemic Stroke Hospital Admissions, Treatment, and Outcomes in Poland in 2009–2013
Source: Front Neurol. 2018 Mar 13;9:134. doi: 10.3389/fneur.2018.00134 (PMC5858531; doi:10.3389/fneur.2018.00134)
Supplement: Supplementary file 1 [file table_1.docx]

Table I. Crude and standardized rates for hospital admissions and 1-year mortality by year of hospitalization, 5-year age bands and sex.

|  |  | **Hospitalizations** | | | | | | | | | **1-year mortality** | | | | | | | | |
| --- | --- | --- | --- | --- | --- | --- | --- | --- | --- | --- | --- | --- | --- | --- | --- | --- | --- | --- | --- |
|  |  | **N** | | | **CAR** | | | **SAR*** | | | **N** | | | **CDR** | | | **SDR** | | |
| **Year** | **Age** | **M** | **F** | **All** | **M** | **F** | **All** | **M** | **F** | **All** | **M** | **F** | **All** | **M** | **F** | **All** | **M** | **F** | **All** |
| **2009** | **30-34** | 87 | 82 | 169 | 5.4 | 5.3 | 5.3 | 4.8 | 5.1 | 5.0 | 10 | 2 | 12 | 0.6 | 0.1 | 0.4 | 0.6 | 0.1 | 0.4 |
|  | **35-39** | 165 | 125 | 290 | 11.2 | 8.7 | 10.0 | 10.2 | 8.6 | 9.5 | 17 | 5 | 22 | 1.2 | 0.3 | 0.8 | 1.0 | 0.3 | 0.7 |
|  | **40-44** | 325 | 204 | 529 | 26.4 | 16.9 | 21.7 | 26.3 | 18.2 | 22.5 | 34 | 17 | 51 | 2.8 | 1.4 | 2.1 | 2.8 | 1.5 | 2.2 |
|  | **45-49** | 782 | 545 | 1327 | 65.4 | 45.7 | 55.5 | 61.4 | 45.9 | 54.0 | 78 | 44 | 122 | 6.5 | 3.7 | 5.1 | 6.1 | 3.7 | 5.0 |
|  | **50-54** | 2131 | 1127 | 3258 | 155.6 | 79.6 | 117.0 | 113.6 | 60.0 | 86.8 | 285 | 103 | 388 | 20.8 | 7.3 | 13.9 | 15.2 | 5.5 | 10.3 |
|  | **55-59** | 3829 | 1804 | 5633 | 272.7 | 118.5 | 192.5 | 164.5 | 70.3 | 115.1 | 557 | 197 | 754 | 39.7 | 12.9 | 25.8 | 23.9 | 7.7 | 15.4 |
|  | **60-64** | 4661 | 2444 | 7105 | 400.5 | 180.9 | 282.5 | 238.2 | 98.9 | 160.8 | 824 | 401 | 1225 | 70.8 | 29.7 | 48.7 | 42.1 | 16.2 | 27.7 |
|  | **65-69** | 4246 | 2846 | 7092 | 628.5 | 330.3 | 461.4 | 512.6 | 225.3 | 341.8 | 940 | 602 | 1542 | 139.1 | 69.9 | 100.3 | 113.5 | 47.7 | 74.3 |
|  | **70-74** | 5333 | 4916 | 10249 | 1006.7 | 632.9 | 784.5 | 781.8 | 357.6 | 510.5 | 1464 | 1324 | 2788 | 276.4 | 170.5 | 213.4 | 214.6 | 96.3 | 138.9 |
|  | **75-79** | 5531 | 7682 | 13213 | 1303.2 | 1066.6 | 1154.3 | 868.8 | 446.9 | 589.7 | 1976 | 2638 | 4614 | 465.6 | 366.3 | 403.1 | 310.4 | 153.5 | 205.9 |
|  | **80-84** | 4237 | 8184 | 12421 | 1521.8 | 1445.6 | 1470.7 | 925.8 | 461.4 | 609.6 | 1985 | 3768 | 5753 | 712.9 | 665.6 | 681.2 | 433.7 | 212.4 | 282.4 |
|  | **85-89** | 2059 | 5604 | 7663 | 1787.1 | 1799.7 | 1796.3 | 1270.4 | 504.9 | 712.7 | 1209 | 3222 | 4431 | 1049.4 | 1034.7 | 1038.7 | 746.0 | 290.3 | 412.1 |
|  | **90-94** | 420 | 1513 | 1933 | 1470.3 | 1658.6 | 1613.7 | 1437.2 | 541.5 | 777.3 | 301 | 1028 | 1329 | 1053.7 | 1126.9 | 1109.5 | 1030.0 | 367.9 | 534.4 |
|  | **30-94** | 33806 | 37076 | 70882 | 240.0 | 239.9 | 239.9 | 209.3 | 137.9 | 169.3 | 9 680 | 13 351 | 23 031 | 52.0 | 67.2 | 59.9 | 36.3 | 26.1 | 30.5 |
| **continued** |  | **Hospitalizations** | | | | | | | | | **1-year mortality** | | | | | | | | |
|  |  | **N** | | | **CAR** | | | **SAR** | | | **N** | | | **CDR** | | | **SDR** | | |
| **Year** | **Age** | **M** | **F** | **All** | **M** | **F** | **All** | **M** | **F** | **All** | **M** | **F** | **All** | **M** | **F** | **All** | **M** | **F** | **All** |
| **2010** | **30-34** | 89 | 91 | 180 | 5.6 | 5.8 | 5.7 | 4.9 | 5.7 | 5.3 | 2 | 3 | 5 | 0.1 | 0.2 | 0.2 | 0.1 | 0.2 | 0.1 |
|  | **35-39** | 157 | 121 | 278 | 10.7 | 8.4 | 9.6 | 9.7 | 8.4 | 9.1 | 18 | 5 | 23 | 1.2 | 0.3 | 0.8 | 1.1 | 0.3 | 0.8 |
|  | **40-44** | 324 | 222 | 546 | 26.3 | 18.4 | 22.4 | 26.2 | 19.8 | 23.2 | 34 | 10 | 44 | 2.8 | 0.8 | 1.8 | 2.8 | 0.9 | 1.9 |
|  | **45-49** | 799 | 506 | 1305 | 66.8 | 42.4 | 54.6 | 62.7 | 42.6 | 53.1 | 83 | 36 | 119 | 6.9 | 3.0 | 5.0 | 6.5 | 3.0 | 4.8 |
|  | **50-54** | 2021 | 1066 | 3087 | 147.6 | 75.3 | 110.9 | 107.7 | 56.8 | 82.3 | 231 | 100 | 331 | 16.9 | 7.1 | 11.9 | 12.3 | 5.3 | 8.8 |
|  | **55-59** | 3863 | 1817 | 5680 | 275.1 | 119.4 | 194.1 | 165.9 | 70.8 | 116.1 | 603 | 236 | 839 | 42.9 | 15.5 | 28.7 | 25.9 | 9.2 | 17.1 |
|  | **60-64** | 4988 | 2603 | 7591 | 428.5 | 192.7 | 301.9 | 254.9 | 105.4 | 171.8 | 864 | 412 | 1276 | 74.2 | 30.5 | 50.7 | 44.2 | 16.7 | 28.9 |
|  | **65-69** | 4171 | 2736 | 6907 | 617.4 | 317.6 | 449.3 | 503.5 | 216.6 | 332.9 | 948 | 565 | 1513 | 140.3 | 65.6 | 98.4 | 114.4 | 44.7 | 72.9 |
|  | **70-74** | 4924 | 4586 | 9510 | 929.5 | 590.4 | 727.9 | 721.8 | 333.6 | 473.7 | 1450 | 1211 | 2661 | 273.7 | 155.9 | 203.7 | 212.6 | 88.1 | 132.5 |
|  | **75-79** | 5132 | 7095 | 12227 | 1209.2 | 985.1 | 1068.2 | 806.1 | 412.8 | 545.7 | 1856 | 2460 | 4316 | 437.3 | 341.5 | 377.1 | 291.5 | 143.1 | 192.6 |
|  | **80-84** | 4039 | 8203 | 12242 | 1450.6 | 1449.0 | 1449.5 | 882.5 | 462.4 | 600.8 | 1886 | 3697 | 5583 | 677.4 | 653.0 | 661.1 | 412.1 | 208.4 | 274.0 |
|  | **85-89** | 2072 | 5922 | 7994 | 1798.4 | 1901.8 | 1873.9 | 1278.4 | 533.6 | 743.5 | 1155 | 3441 | 4596 | 1002.5 | 1105.1 | 1077.4 | 712.6 | 310.0 | 427.5 |
|  | **90-94** | 517 | 1653 | 2170 | 1809.9 | 1812.0 | 1811.5 | 1769.1 | 591.6 | 872.6 | 350 | 1133 | 1483 | 1225.3 | 1242.0 | 1238.0 | 1197.7 | 405.5 | 596.4 |
|  | **30-94** | 33096 | 36621 | 69717 | 233.5 | 235.9 | 234.8 | 200.7 | 133.3 | 163.4 | 9480 | 13309 | 22789 | 50.9 | 67.0 | 59.2 | 35.6 | 25.7 | 30.1 |
| **continued** |  | **Hospitalizations** | | | | | | | | | **1-year mortality** | | | | | | | | |
|  |  | **N** | | | **CAR** | | | **SAR** | | | **N** | | | **CDR** | | | **SDR** | | |
| **Year** | **Age** | **M** | **F** | **All** | **M** | **F** | **All** | **M** | **F** | **All** | **M** | **F** | **All** | **M** | **F** | **All** | **M** | **F** | **All** |
| **2011** | **30-34** | 79 | 83 | 162 | 4.9 | 5.3 | 5.1 | 4.4 | 5.2 | 4.7 | 9 | 6 | 15 | 0.6 | 0.4 | 0.5 | 0.5 | 0.4 | 0.4 |
|  | **35-39** | 171 | 132 | 303 | 11.6 | 9.2 | 10.4 | 10.5 | 9.1 | 9.9 | 19 | 7 | 26 | 1.3 | 0.5 | 0.9 | 1.2 | 0.5 | 0.8 |
|  | **40-44** | 345 | 244 | 589 | 28.0 | 20.2 | 24.1 | 27.9 | 21.8 | 25.1 | 33 | 18 | 51 | 2.7 | 1.5 | 2.1 | 2.7 | 1.6 | 2.2 |
|  | **45-49** | 712 | 442 | 1154 | 59.5 | 37.1 | 48.3 | 55.9 | 37.2 | 47.0 | 71 | 37 | 108 | 5.9 | 3.1 | 4.5 | 5.6 | 3.1 | 4.4 |
|  | **50-54** | 1972 | 1006 | 2978 | 144.0 | 71.1 | 107.0 | 105.1 | 53.6 | 79.4 | 213 | 110 | 323 | 15.6 | 7.8 | 11.6 | 11.4 | 5.9 | 8.6 |
|  | **55-59** | 3851 | 1713 | 5564 | 274.3 | 112.5 | 190.1 | 165.4 | 66.8 | 113.7 | 552 | 222 | 774 | 39.3 | 14.6 | 26.4 | 23.7 | 8.7 | 15.8 |
|  | **60-64** | 5530 | 2752 | 8282 | 475.1 | 203.7 | 329.4 | 282.7 | 111.4 | 187.4 | 927 | 435 | 1362 | 79.6 | 32.2 | 54.2 | 47.4 | 17.6 | 30.8 |
|  | **65-69** | 4367 | 2826 | 7193 | 646.4 | 328.0 | 467.9 | 527.2 | 223.7 | 346.6 | 966 | 601 | 1567 | 143.0 | 69.8 | 101.9 | 116.6 | 47.6 | 75.5 |
|  | **70-74** | 4833 | 4424 | 9257 | 912.3 | 569.6 | 708.6 | 708.5 | 321.8 | 461.1 | 1322 | 1090 | 2412 | 249.6 | 140.3 | 184.6 | 193.8 | 79.3 | 120.1 |
|  | **75-79** | 5177 | 6956 | 12133 | 1219.8 | 965.8 | 1060.0 | 813.2 | 404.7 | 541.5 | 1773 | 2313 | 4086 | 417.8 | 321.1 | 357.0 | 278.5 | 134.6 | 182.3 |
|  | **80-84** | 4386 | 8460 | 12846 | 1575.3 | 1494.4 | 1521.0 | 958.4 | 476.9 | 630.5 | 1944 | 3783 | 5727 | 698.2 | 668.2 | 678.1 | 424.8 | 213.3 | 281.1 |
|  | **85-89** | 2188 | 6422 | 8610 | 1899.1 | 2062.4 | 2018.3 | 1350.0 | 578.6 | 800.8 | 1238 | 3595 | 4833 | 1074.5 | 1154.5 | 1132.9 | 763.9 | 323.9 | 449.5 |
|  | **90-94** | 610 | 2057 | 2667 | 2135.5 | 2254.9 | 2226.4 | 2087.3 | 736.2 | 1072.5 | 392 | 1423 | 1815 | 1372.3 | 1559.9 | 1515.2 | 1341.4 | 509.3 | 729.9 |
|  | **30-94** | 34221 | 37517 | 71738 | 237.4 | 239.3 | 238.4 | 200.2 | 132.0 | 162.6 | 9459 | 13640 | 23099 | 50.8 | 68.7 | 60.0 | 35.5 | 26.2 | 30.3 |
| **Continued** | **Hospitalizations** | | | | | | | | | | **1-year mortality** | | | | | | | | |
|  |  | **N** | | | **CAR** | | | **SAR** | | | **N** | | | **CDR** | | | **SDR** | | |
| **Year** | **Age** | **M** | **F** | **All** | **M** | **F** | **All** | **M** | **F** | **All** | **M** | **F** | **All** | **M** | **F** | **All** | **M** | **F** | **All** |
| **2012** | **30-34** | 83 | 100 | 183 | 5.2 | 6.4 | 5.8 | 4.6 | 6.2 | 5.4 | 4 | 4 | 8 | 0.2 | 0.3 | 0.3 | 0.2 | 0.2 | 0.2 |
|  | **35-39** | 197 | 154 | 351 | 13.4 | 10.7 | 12.1 | 12.1 | 10.6 | 11.5 | 11 | 8 | 19 | 0.7 | 0.6 | 0.7 | 0.7 | 0.6 | 0.6 |
|  | **40-44** | 348 | 262 | 610 | 28.3 | 21.7 | 25.0 | 28.2 | 23.4 | 26.0 | 23 | 11 | 34 | 1.9 | 0.9 | 1.4 | 1.9 | 1.0 | 1.4 |
|  | **45-49** | 740 | 415 | 1155 | 61.8 | 34.8 | 48.3 | 58.1 | 35.0 | 47.0 | 82 | 32 | 114 | 6.9 | 2.7 | 4.8 | 6.4 | 2.7 | 4.6 |
|  | **50-54** | 1785 | 927 | 2712 | 130.4 | 65.5 | 97.4 | 95.2 | 49.4 | 72.3 | 208 | 92 | 300 | 15.2 | 6.5 | 10.8 | 11.1 | 4.9 | 8.0 |
|  | **55-59** | 3919 | 1662 | 5581 | 279.1 | 109.2 | 190.7 | 168.3 | 64.8 | 114.1 | 509 | 212 | 721 | 36.2 | 13.9 | 24.6 | 21.9 | 8.3 | 14.7 |
|  | **60-64** | 5577 | 2795 | 8372 | 479.2 | 206.9 | 332.9 | 285.1 | 113.2 | 189.5 | 927 | 447 | 1374 | 79.6 | 33.1 | 54.6 | 47.4 | 18.1 | 31.1 |
|  | **65-69** | 4831 | 3055 | 7886 | 715.1 | 354.6 | 513.0 | 583.2 | 241.9 | 380.0 | 1029 | 621 | 1650 | 152.3 | 72.1 | 107.3 | 124.2 | 49.2 | 79.5 |
|  | **70-74** | 4858 | 4294 | 9152 | 917.1 | 552.8 | 700.5 | 712.1 | 312.3 | 455.9 | 1304 | 1044 | 2348 | 246.2 | 134.4 | 179.7 | 191.2 | 75.9 | 117.0 |
|  | **75-79** | 5203 | 6627 | 11830 | 1225.9 | 920.1 | 1033.5 | 817.3 | 385.5 | 527.9 | 1867 | 2172 | 4039 | 439.9 | 301.6 | 352.9 | 293.3 | 126.4 | 180.3 |
|  | **80-84** | 4333 | 8393 | 12726 | 1556.2 | 1482.5 | 1506.8 | 946.8 | 473.2 | 624.6 | 1941 | 3621 | 5562 | 697.1 | 639.6 | 658.6 | 424.1 | 204.1 | 273.0 |
|  | **85-89** | 2262 | 6567 | 8829 | 1963.3 | 2108.9 | 2069.6 | 1395.7 | 591.7 | 821.2 | 1252 | 3661 | 4913 | 1086.7 | 1175.7 | 1151.7 | 772.5 | 329.9 | 456.9 |
|  | **90-94** | 631 | 2401 | 3032 | 2209.0 | 2632.0 | 2531.1 | 2159.2 | 859.3 | 1219.3 | 444 | 1648 | 2092 | 1554.3 | 1806.6 | 1746.4 | 1519.3 | 589.8 | 841.3 |
|  | **30-94** | 34767 | 37652 | 72419 | 240.3 | 239.1 | 239.7 | 198.9 | 129.7 | 160.9 | 9601 | 13573 | 23174 | 51.6 | 68.4 | 60.2 | 36.1 | 25.9 | 30.4 |
| **continued** |  | **Hospitalizations** | | | | | | | | | **1-year mortality** | | | | | | | | |
|  |  | **N** | | | **CAR** | | | **SAR** | | | **N** | | | **CDR** | | | **SDR** | | |
| **Year** | **Age** | **M** | **F** | **All** | **M** | **F** | **All** | **M** | **F** | **All** | **M** | **F** | **All** | **M** | **F** | **All** | **M** | **F** | **All** |
| **2013** | **30-34** | 94 | 91 | 185 | 5.9 | 5.8 | 5.9 | 5.2 | 5.7 | 5.4 | 9 | 0 | 9 | 0.6 | 0.0 | 0.3 | 0.5 | 0.0 | 0.3 |
|  | **35-39** | 210 | 153 | 363 | 14.3 | 10.7 | 12.5 | 12.9 | 10.6 | 11.8 | 18 | 5 | 23 | 1.2 | 0.3 | 0.8 | 1.1 | 0.3 | 0.8 |
|  | **40-44** | 342 | 208 | 550 | 27.8 | 17.2 | 22.5 | 27.7 | 18.6 | 23.4 | 19 | 6 | 25 | 1.5 | 0.5 | 1.0 | 1.5 | 0.5 | 1.1 |
|  | **45-49** | 722 | 436 | 1158 | 60.3 | 36.5 | 48.5 | 56.7 | 36.7 | 47.1 | 63 | 31 | 94 | 5.3 | 2.6 | 3.9 | 4.9 | 2.6 | 3.8 |
|  | **50-54** | 1757 | 819 | 2576 | 128.3 | 57.9 | 92.5 | 93.7 | 43.6 | 68.6 | 183 | 79 | 262 | 13.4 | 5.6 | 9.4 | 9.8 | 4.2 | 7.0 |
|  | **55-59** | 3670 | 1697 | 5367 | 261.4 | 111.5 | 183.4 | 157.7 | 66.2 | 109.7 | 506 | 190 | 696 | 36.0 | 12.5 | 23.8 | 21.7 | 7.4 | 14.2 |
|  | **60-64** | 5436 | 2787 | 8223 | 467.0 | 206.3 | 327.0 | 277.8 | 112.8 | 186.1 | 884 | 415 | 1299 | 75.9 | 30.7 | 51.7 | 45.2 | 16.8 | 29.4 |
|  | **65-69** | 5293 | 3357 | 8650 | 783.5 | 389.6 | 562.7 | 639.0 | 265.8 | 416.9 | 1128 | 652 | 1780 | 167.0 | 75.7 | 115.8 | 136.2 | 51.6 | 85.8 |
|  | **70-74** | 4614 | 4030 | 8644 | 871.0 | 518.8 | 661.6 | 676.4 | 293.1 | 430.6 | 1232 | 991 | 2223 | 232.6 | 127.6 | 170.2 | 180.6 | 72.1 | 110.7 |
|  | **75-79** | 5011 | 6425 | 11436 | 1180.7 | 892.1 | 999.1 | 787.1 | 373.8 | 510.4 | 1686 | 2100 | 3786 | 397.3 | 291.6 | 330.8 | 264.8 | 122.2 | 169.0 |
|  | **80-84** | 4243 | 8230 | 12473 | 1523.9 | 1453.7 | 1476.9 | 927.1 | 464.0 | 612.2 | 1815 | 3572 | 5387 | 651.9 | 631.0 | 637.9 | 396.6 | 201.4 | 264.4 |
|  | **85-89** | 2309 | 6613 | 8922 | 2004.1 | 2123.7 | 2091.4 | 1424.7 | 595.8 | 829.8 | 1241 | 3698 | 4939 | 1077.1 | 1187.6 | 1157.8 | 765.7 | 333.2 | 459.4 |
|  | **90-94** | 760 | 2762 | 3522 | 2660.6 | 3027.7 | 2940.2 | 2600.6 | 988.5 | 1416.3 | 529 | 1892 | 2421 | 1851.9 | 2074.0 | 2021.1 | 1810.2 | 677.1 | 973.6 |
|  | **30-94** | 34461 | 37608 | 72069 | 237.5 | 238.2 | 237.9 | 193.0 | 126.6 | 156.7 | 9313 | 13631 | 22944 | 50.0 | 68.7 | 59.6 | 35.3 | 25.8 | 30.0 |

M – male

F – female

CAR – crude annual hospitalization rate (/100,000)

SAR – standardized for WHO world population annual hospitalization rate

CDR – crude annual death rate

SDR – standardized for WHO world population annual death rate

* - SAR in 2013 was lower than in 2009 for males and females (p for trend<0.001).
